# Influence of *Triatoma dimidiata* in Modulating the Virulence of *Trypanosoma cruzi* Mexican Strains

**DOI:** 10.1155/2012/328091

**Published:** 2012-12-03

**Authors:** E. Guzman-Marin, M. Jimenez-Coello, M. Puerto-Solis, A. Ortega-Pacheco, K. Y. Acosta-Viana

**Affiliations:** ^1^Cuerpo Académico Biomedicina de Enfermedades Infecciosas y Parasitarias, CIR “Dr. Hideyo Noguchi”, Universidad Autónoma de Yucatán, Avenida Itzaés No. 490 x Calle 59, 97000 Mérida, YUC, Mexico; ^2^Departamento de Salud Animal y Medicina Preventiva, Facultad de Medicina Veterinaria y Zootecnia, Campus de Ciencias Biologicas y Agropecuarias, Universidad Autonoma de Yucatan, AP 4-116 Merida, YUC, Mexico

## Abstract

The epidemiology of Chagas disease is complex. There are different vectors and reservoirs and different clinical manifestations. In order to assess whether the biological behavior of three strains isolated in southeastern Mexico (H4 isolated from human, Z17 isolated from *Didelphis *sp., and V isolated from *T. dimidiata*) could be modified during passage through the vector *T. dimidiata*, the parasitemia curve, the amount of amastigote nests, and mortality of BALB/c infected with blood trypomastigotes of *T. cruzi* were evaluated. Strains were maintained in continuous passage from mouse to mouse and in animals infected with metacyclic trypomastigotes. The parasitemia curves were significantly different (*P* < 0.05) between mice to mice and triatoma to mice groups in strains H4 and Z17, and was also observed fewer amastigote nests in cardiac tissue (*P* < 0.05 strain H4 with higher number versus all groups and Z17 between mice to mice and triatoma to mice) 45 days after inoculation. It is concluded that *T. dimidiata *influences in modulating the virulence of strains of *T. cruzi *in the region. Further studies of the intestinal tract of the insect in search for some protein molecules involved in regulating may clarify the virulence of the parasite.

## 1. Introduction

American Trypanosomiasis also known as Chagas' disease remains a public health problem throughout Latin America, where it is estimated that 10 million people are infected with *Trypanosoma cruzi* (*T. cruzi*), and other 25 million are at risk to become infected [1].

The causative agent, *T. cruzi*, has a natural biological pleomorphism with biochemical and molecular differences between isolates, showing great differences in their behavior from both *in vitro* and *in vivo* studies that may explain the large variation in the clinical presentations of the disease [2–4]. These differences have been attributed to many causes such as environmental factors, host immunity, virulence, pathogenicity, and passage through various species of vectors and hosts. Those differences need to be characterized from the clinical-epidemiological and clinical-pathological points of view [4–6].

In Mexico, the heterogeneity among the locally isolated strains has been reported, and differences in the onset and severity of the disease in human hosts have been attributed to the susceptibility to infection, to the parasite, and/or vector [7, 8].

In Yucatan, several studies on the biological behavior of isolated *T. cruzi* strains from human cases and reservoirs have been performed. Entomological, epidemiological aspects, and biological behavior of the vectors have also been conducted [4, 9–11].

Based on data obtained from these studies, it has been determined that *Triatoma dimidiata* (*T. dimidiata*) is the main and effective transmitter of *T. cruzi* in the endemic region of Yucatan, Mexico, and that the presence of different infected reservoirs including humans contributes to the maintenance of Chagas' disease. However, despite being present the epidemiological conditions for the transmission to humans, only 42 cases were diagnosed with Chagas' cardiomyopathy in acute and chronic stages from 1970 to 1995 [12, 13]. This is a low number of clinical cases compared to the situation in other states of Mexico and other endemic regions of the world. These may be attributed to various reasons such as changes in the virulence of the parasite as it passes through the vectors, the Mayan population resistance to infection, or lack of accurate epidemiological studies.

The objective of this study was to determine the role of *T. dimidiata* modulating the virulence of *T. cruzi* which may explain in part the clinical and epidemiological features of the disease in humans from Yucatan.

## 2. Material and Methods

### 2.1. Parasites

Three strains of *T. cruzi* isolated in the State of Yucatan, Mexico, were used: H4 (isolated from human), Z17 (isolated from the reservoir *Didelphis marsupialis*), and V (isolated from the vector *Triatoma dimidiata maculipennis*).

All isolates were maintained by successive passages through mouse to mouse (control group) and *Triatoma* to mouse (experimental group) for their biological characterization.

### 2.2. Insects

Three groups of five 5th stage nymphs of *T. dimidiata* were used for the triatoma-mouse passes for each strain. This stage was chosen because when insects feed under laboratory conditions, the 4th and 5th stage nymphs feed for longer, and have higher intake capacity, ensuring the capture of the parasite. For the infection of triatomines, mice infected with each of the strains (maintained by successive passages of mouse-mouse) were previously anesthetized with sodium pentothal (40 mg/Kg) intraperitoneally (IP) and kept in a box along with triatomines to feed them for 15 to 30 min. After feeding, triatomines were placed in a jar for 30 to 60 days to become parasitized. Once parasitized, triatomines were compressed in the abdomen to get all the feces and search for parasites; thus, there were obtained inoculums of trypomastigotes to infect each mouse of the experimental groups. Triatomines infected with the different isolates of *T. cruzi* (H4, Z17, and V) were kept for 60 days and then used for the infection of mice from the experimental groups denominated, triatoma to mice.

### 2.3. Animal Model

A total of 180 BALB/c 35 g, 8 weeks of age male mice were used. BALB/c mice were maintained on a 12 : 12 h light-dark cycle and had access to food and water *adlibitum*.

### 2.4. Experimental Design

Mice were divided into a total of 18 groups: 9 groups for the experimental condition “mice to mice” and 9 groups to evaluate triatoma to mice groups. Each group included 10 animals. There were 3 strains evaluated, H4, Z17, and V. Each strain was evaluated by triplicated. In the mice to mice groups, *T. cruzi *inoculums were obtained from isolates maintained by passages mouse to mouse. From the triatoma to mice groups, the inoculums were obtained from isolates maintained on passes mouse to triatoma and triatoma to mouse. 

Three groups of mice were inoculated with a different isolate H4 (human), Z17 (*Didelphis* sp.), and V (Vector *T. dimidiata*), directly in passage mice to mice, in the same way 3 groups of mice were inoculated with each different isolate. For the infection of mice to mice groups, twenty days after inoculation, mice were bled to obtain an inoculum of 3 × 10^5^ parasites/mL blood trypomastigotes of each of the isolates of *T. cruzi* to study, and for triatoma-mice groups infection, the number of trypomastigotes obtained from infected *T. dimidiata* dejections was adjusted to a final concentration of 3 × 10^5^ parasites/mL with a solution of NaCl (0.85%) and EDTA (0.05%), mixed, and then counted on Neubauer chamber. Each mouse from the 18 groups formed inoculated IP with 200 *μ*L of the mixture [6, 14].

### 2.5. Biological Characterization

The virulence was evaluated for each experimental and control group in terms of parasitemia curves, tissue invasion, and mortality rate from the infected mice groups. To determine the parasitemia level, the number of blood trypomastigotes was quantified in a Neubauer chamber. A volume of 0.5 mL of peripheral blood complete with 0.85% saline and with 0.05% EDTA was taken from the tail of each mouse. The parasite count was performed every 4 days until the disappearance of parasitemia. 

The invasion of tissues was determined by the presence of amastigote nests in different tissue organs evaluated (heart, skeletal muscle, liver, and spleen) and obtained after euthanasia of the mice at day 45 postinfection. Tissue samples were fixed in 10% formalin and processed using histological sections of 5 *μ*m thick each (5 slices per organ); samples were later stained with hematoxylin-eosin (HE) and evaluated in search and quantified of amastigote nests with the 40x objective. Mortality rate was evaluated considering the percentage of spontaneously dead mice during all period of the study.

### 2.6. Data Analysis

Data obtained from the biological behavior (curves of parasitemia and amastigote nests and mortality) between the experimental and control groups were analyzed with statistical *t*-Student test and one-way ANOVA with the Tukey posttest. The significance value *P* < 0.05 determines significant differences [15, 16]. The mortality of mouse after inoculation of the strains was analyzed using a life table from the software SPSS v 16. For comparison between groups (mouse-mouse and triatoma-mouse), the logrank test was used. 

## 3. Results

**Figure 1:**
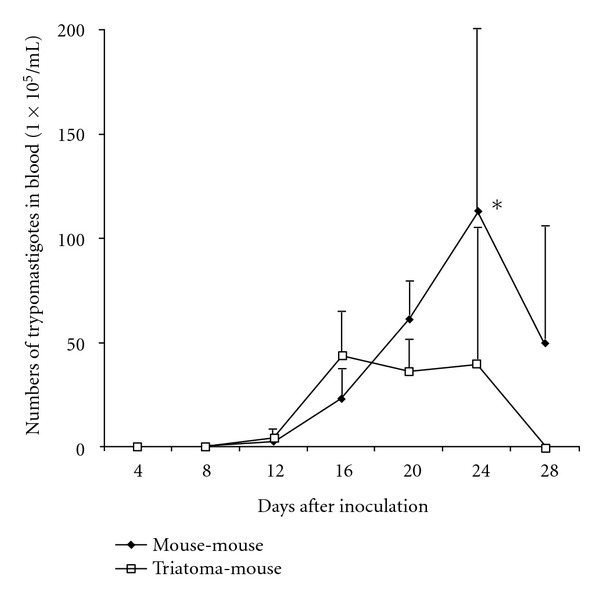
Parasitemia curve of H4 strain (mouse-mouse) versus (triatoma-mouse). (*P value < 0.05 in comparison with all evaluated groups).

In the H4 strain mouse-mouse, parasitemia started on day 8 postinfection, reaching a peak on day 24 with a parasitemia load of 115 × 10^5^ parasites/mL; the parasitemia level showed statistically significant difference in comparison with the other evaluated groups (*P* < 0.05). In the case of the H4 strain triatoma-mouse, the parasitemia began on day 8 with a peak on day 14 when the parasitemia load was 44 parasites/mL (Figure 1).

**Figure 2:**
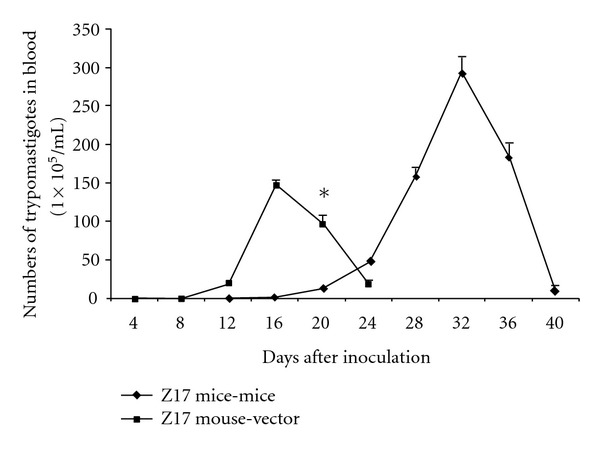
Parasitemia curve of strain Z17 (mouse-mouse versus triatoma-mouse). *P value < 0.05 in comparison between both groups.

The infection with Z17 strain, from mouse to mouse parasitemia began on day 12 and peaked at day 32 postinfection with a parasite load of 295 × 10^5^ parasites/mL, for the triatoma-mouse group, the parasitemia began on day 8 reaching a the peak on day 13 postinfection with a parasite load of 149 × 10^5^ parasites/mL (Figure 2) and showed a statistically significant difference when was compared with the Z17 mouse-mouse group at day 20 postinfection (*P* < 0.05), but the duration of the parasitemia of the group Z17 triatoma to mouse was shorter than mouse to mouse.

**Figure 3:**
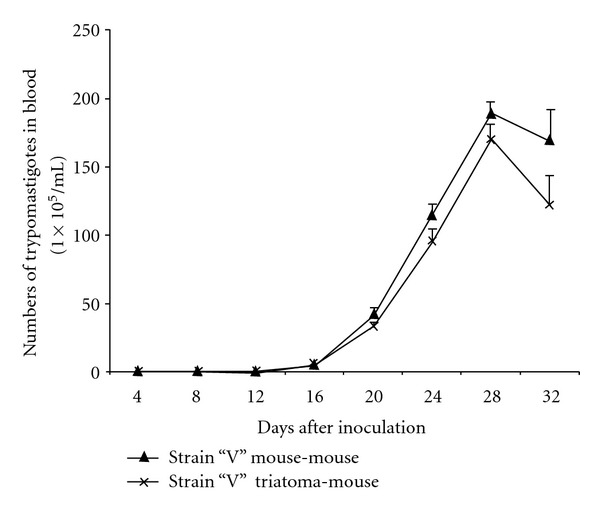
Parasitemia curve of strain “V” (mouse-mouse versus triatoma-mouse).

For the V strain, both mouse-mouse and triatoma-mouse groups, parasitemia starts on day 12 postinfection with the peak on day 28. The parasitemia load postinfection from mouse to mouse was 190 × 10^5^ parasites/mL, and the parasitemia load from triatoma-mouse was 170 parasites/mL (Figure 3).

The virulence of the studied strains (H4, Z17, and V) began between day 8 and 12 with peaks of 115, 295, and 190 × 10^5^ parasites/mL in the mice to mice groups and between on 12 and 32, with peaks of 44, 149, and 170 × 10^5^ parasites/mL in the triatoma to mice groups. Regarding the parasitemia, a significant difference between mice to mice and triatoma to mice groups infected with the strains H4 and Z17. On the other hand, for V strain, the *P*  value was not significant (0.06). 

**Figure 4:**
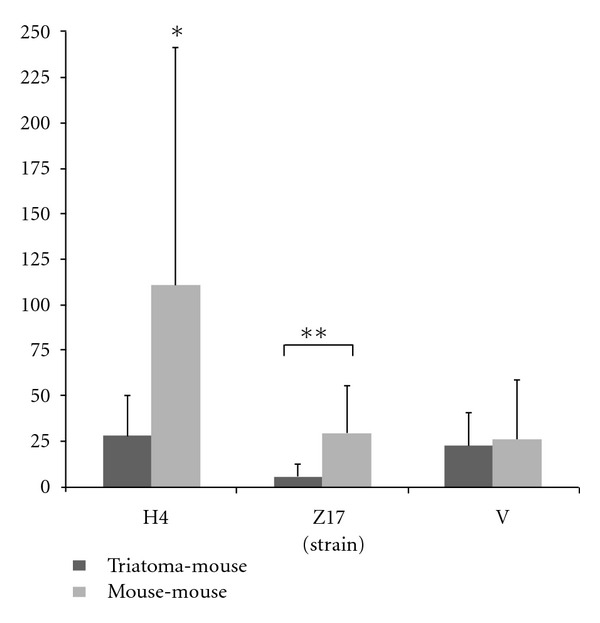
Number of amastigote nests from *T. cruzi* in 100 fields, 40x of cardiac tissue from infected BALB/c mice with the H4, Z17, and V strains (*P < 0.05 in comparison with all evaluated groups. **P < 0.05 in comparison between both groups).

Amastigote nests were only observed on the cardiac tissue. When comparing the three strains (H4, Z17, and V), the H4 strain mouse-mouse produced the greatest number of nests (<0.05 in comparison with all evaluated groups). In the groups triatoma-mouse, from strains H4 and V, the number of amastigote nests were 28 and 22, respectively, and for the Z17 strain, were counted only 5 nests. This reduction was statistically different (<0.05) when compared between mouse to mouse group versus triatoma to mouse group (Figure 4).

**Figure 5:**
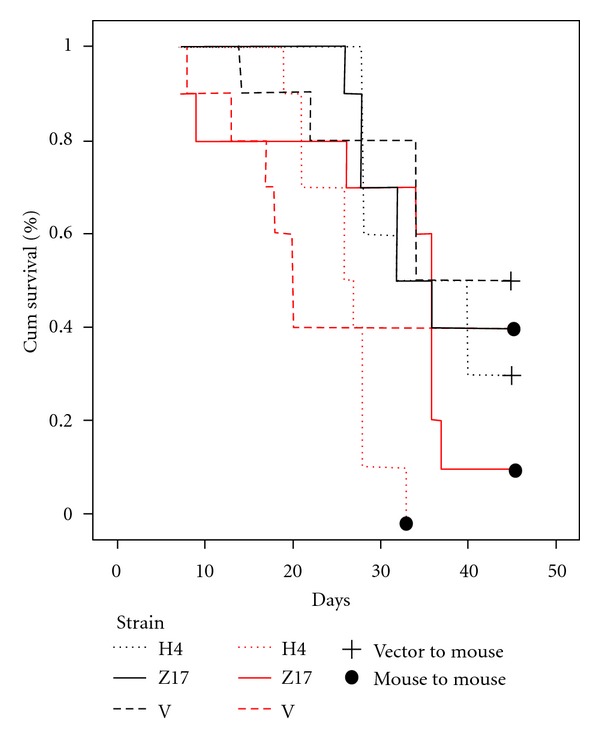
Survival rate of *T. cruzi *strains H4, Z17, and V in BALB/c mice infected “mice to mice” and “triatoma to mice” during 45 days.

The mortality recorded for the H4 strain was 70% (mouse-mouse) and 100% (triatoma-mouse), for the Z17 strain, 60% (mouse-mouse) and 90% (triatoma-mouse), and for the strain V, 50% (mouse-mouse) and 80% (triatoma-mouse). A significant difference on the survival analysis was found (*P* < 0.05). Mortality occurred faster in the strains H4 and Z17 from the mouse-mouse groups (Figure 5).

## 4. Discussion

Chagas disease is an important endemic protozoa infection in Mexico. One of the main pathologic characteristic, as seen in other countries, is the cardiac damage. However, despite that in Yucatan the vector is widely distributed and the parasite is present in several reservoir mammals, there is no important number of clinical reports from cardiomyopathies or fatal cases in the region even when there are no official strategies for the control of the vector. 

The clones H4, Z17, and V isolated from the Yucatan area showed a remarkable difference in the parasitemia level when the parasites from the inoculums were passed through the vector *T. dimidiata. *It should be noted that the biological, biochemical, and molecular strains of *T. cruzi* have been characterized by several authors so that each strain sharing these aspects could be representative of a single group. Due to the heterogeneity of the strains, *T. cruzi* has a natural pleomorphism responsible of the severity of the disease when present in the human host and other mammals [5, 17]. 

Current research has shown that biological and biochemical characteristics of three strains (19 SF, 21 SF, and 22 SF) were unchanged after its passage through three different species of triatomines. The maintenance of their biochemical characteristics indicates that virulence may be a variable parameter, but the genetic patterns are stable phenotype [18]. 

The presence of different strains of the parasite found from vectors, reservoirs, and regions from the countries may be responsible of the levels of parasitemia and invasion of certain organs. This variation on their behavior may be due to several reasons such as environmental factors, immunity, virulence, pathogenicity, and deferential step transmitters and host species [3, 19]. 

In the present study the parasitemia and its duration were reduced in the triatoma-mouse group similarly as described by Vallejo et al. [20], which states that the interactions of subpopulations of the trypanosomes with different species and populations of Triatominae determine the epidemiology of the human-infecting trypanosomes in Latin America.

The mortality rates from the studied strains in mice experimentally infected with metacyclic parasites (obtained from a vector) from the southeast of Mexico are higher than others previously reported in the occident of Mexico [8]. The lower velocity of mortality observed in the triatoma-mouse groups may indicate a lower virulence when compared with mouse-mouse H4 and Z17 strains of *T. cruzi*.

A Bolivia reference strain (strain Bolivia) of *Trypanosoma cruzi* (*T. cruzi*) demonstrates clear muscular tissue tropism, however different clones of the same may have affinity for different organs may migrate to produce skeletal muscle or cardiac disorders [21]. Similarly Mexican strains have shown tropism for skeletal and cardiac muscle [8] in coincidence with the ones studied in this work, however these characterizations were made in direct passes and should be noted that the behavior changes (mainly in the intensity of virulence) when the parasite passes through the vector as demonstrated in this study. It would be important to know the behavior of different clones of these strains to evaluate their behavior as they pass through the vector [22].

A study comparing *T. cruzi* strains from Mexico with other Mexican strains show significant differences in several biological parameters by Gómez-Hernández et al., [8], which suggests the intraspecies variations observed in the other Mexican strains might be related to differences in biological behavior *in vivo. *Also was cited that clonal genotypes of *T. cruzi* differ significantly in terms of infectivity, demonstrating an association between genotype diversity, tropism, and pathogenicity [20]. 

However, in the present study, when parasitemia level was compared, a significant difference (*P* < 0.05) between strains H4 and Z17 was found between mouse-mouse and triatoma-mouse groups, and one strain undergoes changes that are not significant (strain V isolated from a vector *T. dimidiate *from the studied area), indicating that the biological behavior of the studied strains was different, and that maybe it could be modified when passing through the *T. dimidiata* vector and their virulence being reduced, explaining partially why the number of human cases of the disease in the Yucatan, Mexico, area is low. However, because of the chronic-degenerative characteristic of the disease, there may be an important amount of cases that are not properly diagnosed or are undiagnosed (persons infected but in the indeterminate phase of the disease, without any evident clinical sign). It is important and interesting to point that perhaps the behavior of certain strains is influenced by the predominant vector in each endemic area, which in this case *T. dimidiata* is the only recognized vector of *T. cruzi* present in the region. This confirms a great adaptation of strains to the only existing vector in the region. In cases of *Leishmania infantum* infection, a virulence deficit occurs by successive *in vitro* passages as a result from an inadequate capacity of the protozoa to differentiate into amastigote forms [22]. This low virulence is a reversible phenomenon, since serial passages on susceptible mice allow the parasite to recover a virulence phenotype. In the case of  *T. cruzi*, when the parasite is in the midgut of the vector, it has been described than the interactions between the parasite with digestive enzymes, hemolysins, agglutinins, and antimicrobial compounds involve a mechanism of defense reactions that maybe responsible of regulating the development of *T. cruzi* in the triatomine vectors gut, and the capability of the parasite to establish an infection could be affected depending the lineage involved [23]; even these evidences have been found in *Rhodnius prolixus*. These interactions may also reduce the virulence of the strains as they pass from vectors and may partially explain the results from the present study.

## 5. Conclusion

This paper demonstrates that the vector *T. dimidiata* may influence in modulating to some degree of the virulence of *T. cruzi* strains isolated from mammals in the southeast of Mexico (Yucatan area), which leads to the need for further studies of the intestinal tract of the vector in search of some protein molecules that interact with the parasite by modulating their virulence. Further studies evaluating and characterizing virulence of *T. cruzi* strains should include real-time PCR from hearth tissue and correlate it with the parasitemia in peripheral blood and heart tissue.
